# Multilevel Zero-One Inflated Beta Regression Model for the Analysis of the Relationship between Exogenous Health Variables and Technical Efficiency in the Spanish National Health System Hospitals

**DOI:** 10.3390/ijerph181910166

**Published:** 2021-09-27

**Authors:** Ricardo Ocaña-Riola, Carmen Pérez-Romero, Mª Isabel Ortega-Díaz, José Jesús Martín-Martín

**Affiliations:** 1 Escuela Andaluza de Salud Pública, Cuesta del Observatorio, 4. Campus Universitario de Cartuja, 18011 Granada, Spain; ricardo.ocana.easp@juntadeandalucia.es (R.O.-R.); carmen.perez.easp@juntadeandalucia.es (C.P.-R.); 2 Instituto de Investigación Biosanitaria ibs.GRANADA, Avda. de Madrid, 15. 18012 Granada, Spain; jmartin@ugr.es; 3 Cátedra de Economía de la Salud y Dirección de Organizaciones Sanitarias (Esalud2) Cuesta del Observatorio, 4. Campus Universitario de Cartuja, 18011 Granada, Spain; 4 Departamento de Economía, Edificio D-3, Campus Las Lagunillas s/n, Universidad de Jaén, 23071 Jaén, Spain; 5 Departamento de Economía Aplicada, Facultad de Ciencias Económicas y Empresariales, Campus Universitario de Cartuja s/n, Universidad de Granada, 18071 Granada, Spain

**Keywords:** efficiency, data envelopment analysis, multilevel zero-one inflated beta regression, public hospitals, private hospitals, public-private partnership, intensive care units, emergency services

## Abstract

Background: This article proposes a methodological innovation in health economics for the second stage analysis of technical efficiency in hospitals. It investigates the relationship between the installed capacity in regions and hospitals and their ownership structure. Methods: A multilevel zero-one inflated beta regression model is employed to model pure technical efficiency more adequately than other models frequently used in econometrics. Results: Compared to publicly managed hospitals, the mean efficiency index of hospitals with public-private partnership (PPP) formulas was 4.27-fold. This figure was 1.90-fold for private hospitals. Concerning the efficiency frontier, the odds ratio (OR) of PPP models vs. public hospitals was 42.06. The OR of private hospitals vs. public hospitals was 8.17. A one standard deviation increase in the percentage of beds in intensive care units increases the odds of being situated on the efficiency frontier by 50%. Conclusions: The proportion of hospital beds in intensive care units relates to a higher chance of being on the efficiency frontier. Hospital ownership structure is related to the mean efficiency index of Spanish National Health Service hospitals, as well as the odds of being situated on the efficiency frontier.

## 1. Introduction

Fluctuations in the demand for healthcare have an impact on the efficiency of hospitals, which has created uncertainty about the optimal use of installed capacity [1,2,3]. This uncertainty is most intense in hospital emergency departments [4,5,6] and emergency healthcare services [6,7]. Both are services on the front line of hospital care, which influences the composition of hospital caseloads [8], and requires a provision of resources which is highly dependent on uncertain fluctuations in demand. This may explain, in part, the observed differences in hospital efficiency [1,9]. Primary care services also play an important role as gatekeepers to the health care system. They indirectly influence the complexity of hospital caseloads and their expenditure levels by avoiding unnecessary and costly hospital diagnostic and therapeutic procedures [10,11].

Traditionally, the literature on hospital efficiency differentiates between the characteristics of hospitals (such as technological provision, teaching nature of the center, rural or urban location, type of ownership) [12,13,14], and those of the environment in which they operate (region, gross domestic product per capita, population density) [13,14,15,16]. However, there is no previous research which analyzes the relationship between hospital efficiency and the characteristics of the emergency department and primary care structure as a whole. Only one study in Germany has evaluated the relationship between emergency departments and the technical efficiency of hospitals [16]. The study used a two-stage double bootstrap data envelopment analysis approach with truncated regression and found a negative relationship between the dispersion of emergency departments, their caseload, and hospital efficiency.

The relationship between hospital efficiency and ownership structure has been studied more recently [17,18]. Studies in European countries have evaluated the technical efficiency of public and private hospitals with data envelopment analysis (DEA) [19,20,21,22,23,24], which has led to ambiguous and contradictory results. The regulatory and management framework to which they are subject presents as more relevant than ownership be it public or private [14,25]. The efficiency of the various public-private partnership (PPP) formulas, which have experienced significant growth in recent years, has also been investigated [26,27,28,29]. PPP is defined as a long-term contract between a private actor and a government agency to provide a public asset or service, in which the private actor assumes the risks and management responsibility [30,31]. The available evidence on the efficiency of PPP models compared to other hospital management structures is limited and contradictory [26,32,33,34,35,36,37,38,39,40,41].

In the literature on hospital efficiency, the predominant approach is a combination of non-parametric frontier models, particularly DEA, with different types of multivariate regressions (Tobit regression, ordinary least squares regression, logistic regression, and truncated regression) employed as second-stage analyses which relate the level of technical efficiency obtained to different hospital characteristics [42,43].

**Figure 1:**
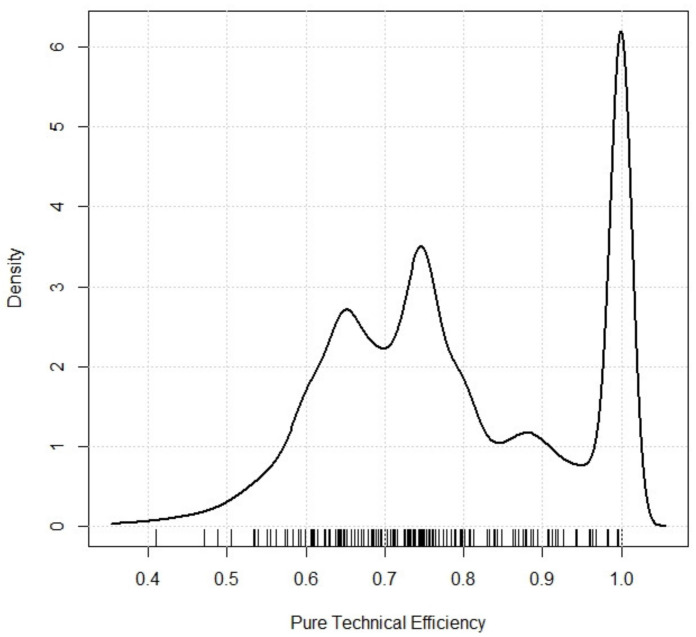
Density function of pure technical efficiency in 173 hospitals in the Spanish National Health System. Source: Prepared by the authors.

Recently, the use of multilevel or hierarchical models, both linear and logistic, has made it possible to incorporate variables from hospital contexts, modelling intra- and inter-level variability. These models allow for the estimation of the effect of explanatory variables (both hospital and contextual), as well as studies of the variability among hospitals and among the contexts in which they are located [13,14,15,44,45,46,47]. However, a common problem is inappropriateness of efficiency scores in a normal distribution function, which can lead to goodness-of-fit problems in the linear model. Pure technical efficiency (PTE) is a continuous variable, with values restricted to the interval (0, 1). Furthermore, the units of analysis that reach the efficiency frontier show accumulation points at value 1 (Figure 1).

These characteristics of PTE mean that its density function, $f\left(PTE\right)$, can be represented by a one-inflated $Beta\left(\alpha_{1},\alpha_{2}\right)$ distribution, $\alpha_{1}>0$ *y* $\alpha_{2}>0$. This density function is integrated in the zero-one inflated family distributions, with the expression:

(1) $$f\left(PTE\right)=\left\{\begin{array}{c} \omega_{01}\left(1-\omega_{1}\right)\;\;\;\;\;\;\;\;\;\;\;\;\;\;\;\;\;\;\;\;\;\;\;\;\;\;\;\;\;\;PTE=0\\ \omega_{01}\omega_{1}\;\;\;\;\;\;\;\;\;\;\;\;\;\;\;\;\;\;\;\;\;\;\;\;\;\;\;\;\;\;\;\;\;\;\;PTE=1\\ \left(1-\omega_{01}\right)f_{Beta}\left(PTE\right)\;\;\;\;\;\;\;\;\;\;\;\;\;\;\;\;\;\;\;\;\;\;0<PTE<1 \end{array}\right\}$$

where $\omega_{01}$ is the probability of zero-one inflation (probability that the efficiency variable takes the value 0 or the value 1), $\omega_{1}$ is the conditional probability of inflation one (probability that the efficiency variable takes the value 1 instead of 0) and $f_{Beta}\left(PTE\right)$ is the density function of the $Beta\left(\alpha_{1},\alpha_{2}\right)$, distribution, with mean $µ=\frac{\alpha_{1}}{\alpha_{1}+\alpha_{2}}$ [48].

This probability distribution is the basis for the zero-one inflated beta regression model, which allows variables of this type to be modelled [49]. The model assumes that the dependent variable has a mixed distribution, consisting of two distributions: a continuous one, described by the beta distribution, and a binary one, with probability mass at zero or one. The density function of the beta distribution can take multiple forms, depending on the two parameters which define it. This flexible quality allows for an optimal data fit. The parameters of the mixture are modelled in terms of the regression coefficients, which can be estimated using maximum likelihood methods or Bayesian inference. Bayesian inference is usually preferred for this type of model due to the complexity of the estimates and the convergence difficulties of the maximum likelihood method [50].

The zero-one inflated beta regression model can be extended to a multilevel model, where the information is clustered hierarchically at several levels. This is a recent line of research in the field of mathematical statistics, which is currently under development. In this paper we have carried out such a multilevel extension, proposing, for the first time, a multilevel zero-one inflated beta regression model for PTE second stage analysis. The use of this model has permitted analysis of two strategic issues in health policy design. Firstly, the investigation of the relationship of hospital efficiency to installed capacity at both the hospital and regional levels to cope with potential sudden increases in demand due to exogenous global health shocks. Secondly, to study how the ownership structure of the centers affects both the level of technical efficiency of the hospital and the probability of being located on the efficiency frontier.

## 2. Materials and Methods

### 2.1. Decision Making Units (DMUs) 

This empirical analysis was performed on a set of general hospitals of public, private, or PPP ownership which make up the Spanish National Health System (SNHS). The exclusion criteria were: having less than 50 beds, not having registered activity in the emergency department and not having information presented on all inputs and outputs considered. In addition, hospitals located in autonomous cities (Ceuta and Melilla) were excluded due to the particularities of these territories. Under these criteria, the group of hospitals analyzed consisted of 173 centers.

### 2.2. Variables

The inputs used for DEA are purchases and external services acquired, installed beds, healthcare personnel and non-health personnel. To avoid larger hospitals being penalized, the purchases and external services variable was standardized and refers to expenditure per bed [51]. The outputs used are total adjusted case-by-case discharges and outpatient activity (Table 1).

As independent variables potentially related to PTE, this study includes a set of exogenous hospital and regional variables (Table 2). In each hospital, the training of specialists and the availability of resources in hospital emergency departments and intensive care units (emergency physicians, beds, and their occupancy) were considered. The ownership of the health centers was also considered, differentiating between public, private, and PPP formulas. Each Spanish region includes two economic variables (public healthcare expenditure and average annual income per household), and a set of variables that characterize the provision of hospital resources (private bed, public general bed, intensive care unit public beds, and emergency physician population rates), primary care (outpatient emergency center, doctor and nurse population rates) and healthcare emergencies (emergency professionals per 1000 inhabitants) available to confront a potential pandemic or health crisis in the future (Appendix A).

### 2.3. Sources of Information

Data on hospitals were obtained from the Specialised Care Information System (SIAE) and the Specialised Care Activity Register (RAE-CMBD), both produced by the Spanish Ministry of Health, Consumption, and Social Welfare as part of the Government of Spain. The rest of the variables included in the analysis were obtained from the system of key indicators of the SNHS provided by the aforementioned Ministry, as well as from the statistics published by the Spanish Institute for National Statistics.

### 2.4. Data Analysis

The data analysis consisted of two phases: analysis of each hospital PTE by means of DEA, and a second-stage analysis to identify exogenous variables related to technical efficiency using a multilevel zero-one inflated beta regression model.

#### 2.4.1. Data Envelopment Analysis

Frontier models base their methodological strategy on the explicit construction of an efficiency frontier. This efficiency frontier is determined by the DMUs whose management is considered unbeatable within the set of all those evaluated from the point of view of relative efficiency. Initially proposed by Charnes et al., mathematical programming was used to construct the technological frontier which represents best practices, and to calculate an efficiency index for each DMU. This efficiency index was obtained by comparing the position of each with respect to the frontier [52,53].

In line with this methodology, this study employs a BCC model, which considers variable returns to scale, to determine the value of the PTE [54], and an output maximization orientation. The latter implies that it would not be possible for efficient DMUs to increase their outputs given the inputs used. The analytical formulation of the output-oriented BCC model is:

Max ϕ

s.t.

$$\overset{n}{\underset{j=1}{\sum }}\lambda_{j}x_{ij}\leq x_{i0}\;\;\;,\;i=1,\ldots ,m$$

$$\overset{n}{\underset{j=1}{\sum }}\lambda_{j}y_{rj}\geq \phi y_{r0}\;\;\;,\;r=1,\ldots ,s$$

$$\overset{n}{\underset{j=1}{\sum }}\lambda_{j}=1$$

$$\lambda_{j}\geq 0$$

This optimization program consists of a vector of *n* DMUs made up of m inputs and s outputs, such that:

$$x_{ij}=\mathrm{quantity}\;\mathrm{of}\;\mathrm{input}\;i\;\mathrm{consumed}\;\mathrm{by}\;\mathrm{DMU}\;j\;(\mathrm{there}\;\mathrm{being}\;n\;\mathrm{DMUs}).$$

$$y_{rj}=\mathrm{quantity}\;\mathrm{of}\;\mathrm{output}\;r\;\mathrm{produced}\;\mathrm{by}\;\mathrm{DMU}\;j.$$

ϕ = proportion by which the outputs can be increased.

$$\lambda_{j}=\mathrm{intensity}\;\mathrm{of}\;\mathrm{DMU}\;j\;\mathrm{in}\;\mathrm{the}\;\mathrm{construction}\;\mathrm{of}\;\mathrm{the}\;\mathrm{reference}\;\mathrm{DMU}.$$

The value of ϕ cannot be less than one and provides information on the technical efficiency of each DMU. Specifically, ϕ−1 represents the proportional increase in outputs which could be obtained by keeping inputs constant. Furthermore, 1/ϕ  represents the relative measurement of pure technical efficiency and has a range between 0 and 1. The DMU is efficient and is located on the production frontier when the resulting value is 1. On the other hand, values lower than 1 indicate that DMUs are inefficient, meaning that, through better input management, output could be increased without changing the amount of inputs used [55].

#### 2.4.2. Second-Stage Analysis

A multilevel zero-one inflated beta regression model was used to study the variables related to PTE. This modelling considers the hierarchical structure of the information, where the hospitals (level 1) are grouped by autonomous communities (level 2). Thus, for each hospital *i* = 1, ..., 173 in the autonomous community *j* = 1, ..., 17, the parameters of the function $f\left(PTE_{ij}\right)$, described in (1), was modelled as follows:

(2) $$logit\left({µ}_{ij}\right)=log\left(\frac{{µ}_{ij}}{1-{µ}_{ij}}\right)=\left(\beta_{0}+u_{0j}\right)+\overset{l^{\left(1\right)}}{\underset{r=1}{\sum }}\beta_{r}^{\left(1\right)}x_{rij}^{\left(1\right)}+\overset{l^{\left(2\right)}}{\underset{r=1}{\sum }}\beta_{r}^{\left(2\right)}x_{rj}^{\left(2\right)}$$

(3) $$logit\left(\omega_{o1ij}\right)=log\left(\frac{\omega_{01ij}}{1-\omega_{01ij}}\right)=\gamma_{0}+\overset{l^{\left(1\right)}}{\underset{r=1}{\sum }}\gamma_{r}^{\left(1\right)}x_{rij}^{\left(1\right)}+\overset{l^{\left(2\right)}}{\underset{r=1}{\sum }}\gamma_{r}^{\left(2\right)}x_{rj}^{\left(2\right)}$$

$$\omega_{1ij}=\theta$$

$$\alpha_{1ij}+\alpha_{2ij}=\varphi$$

where ${µ}_{ij}=\frac{\alpha_{1ij}}{\alpha_{1ij}+\alpha_{2ij}}$ is the mean of the $Beta\left(\alpha_{1ij},\alpha_{2ij}\right)$ distribution for hospital *i* = 1, …, 173 in the region *j* = 1, …, 17. Furthermore, the value $I_{ij}=\frac{{µ}_{ij}}{1-{µ}_{ij}}$ can be interpreted as a mean efficiency index (MEI), where a value greater than 1 indicates that the mean efficiency of the hospital, ${µ}_{ij}$, exceeds the inefficiency, $1-{µ}_{ij}$.

Both the mean of the beta distribution, ${µ}_{ij}$, and the zero-one inflation probability, $\omega_{01ij}$, transformed using the logit function, are modelled through a linear combination of the independent variables $x_{rij}^{\left(1\right)}$ and $x_{rj}^{\left(2\right)}$ representing, respectively, level 1 (*r* = 1, ..., *l*^(1)^) and level 2 (*r* = 1, ..., *l*^(2)^) variables. All quantitative independent variables were standardized to obtain dimensionless variables with mean zero and standard deviation one. The intercept term $\beta_{0j}=\beta_{0}+u_{0j}$ is a random effect whose error, represented by *j* $u_{0j}$, follows a normal distribution with mean 0 and variance $\;\sigma_{u_{0}}^{2}$. The coefficients of the independent variables, represented respectively by $\beta_{r}^{\left(1\right)}$, $\beta_{r}^{\left(2\right)}$, and $\gamma_{r}^{\left(1\right)}$ *y* $\gamma_{r}^{\left(2\right)}$, are fixed effects. The conditional probability of one-inflation, $\omega_{1ij}$, and the precision, $\alpha_{1ij}+\alpha_{2ij}$, remain constant.

The estimation of the model parameters was performed by full Bayesian inference, assuming improper flat prior distributions for all fixed effects and normal distribution for the variance of the random error. In Markov Chain Monte Carlo (MCMC) sampling, 1000 warmup iterations and 5000 subsequent updates were used. Convergence of the estimates was ensured using four chains and a diagnostic using the parameter $\hat{R}$, the bulk effective sample size (Bulk-ESS) and the tail effective sample size (tail-ESS). Convergence was obtained with $\hat{R}<1,01$, Bulk-ESS > 400 and tail-ESS > 400 [56]. Gamma and normal a priori distributions were used for sensitivity analysis [57].

In Bayesian statistics, model coefficients are not considered fixed parameters, but random variables which have a distribution function. Bayesian inference estimates the posterior distribution function of the coefficient, taking the a priori function and the information provided by the data into account. The mean of the posterior distribution function is considered as the coefficient estimate. However, all other values of this function could also be possible values of the coefficient.

The analysis of the a posteriori distribution function of each coefficient yielded the 2.5 and 97.5 percentiles, which formed the credibility interval with 0.95 probability. In addition, the positive coefficient probability (PCP) and the negative coefficient probability (NCP) were used to test the hypothesis of a positive or negative relationship of each independent variable with the dependent variable.

Once the model is estimated, $exp\left(\beta_{r}^{\left(k\right)}\right)$ is the ratio of mean efficiency indices (RMEI) among hospitals which are not on the efficiency frontier and $exp\left(\gamma_{r}^{\left(k\right)}\right)$ is the odds ratio (OR) of being situated on the efficiency frontier, *k* = 1, 2.

## 3. Results

18.5% of the hospitals analyzed (32) were efficient, and are situated on the efficiency frontier. The mean of PTE was 0.78 (78%), i.e., for the individual levels of inputs used, the average increase in output which could be achieved with improved management was 0.22 points (22%).

Within the second-stage analysis, the multilevel zero-one inflated beta regression model showed values of $\hat{R}$ equal to 1 for all coefficients. Furthermore, the effective sample sizes were greater than 400, indicating a good performance of the estimation method in achieving adequate convergence.

The form of hospital management and the number of resident doctors showed coefficients with credible intervals which did not contain the value 0, or had a very high positive coefficient probability in the posterior distribution function (Table 3) (Appendix B). Compared to publicly managed hospitals, the mean efficiency index of the hospitals subject to PPP formulas was 4.27-fold. This value was 1.90-fold in the case of private hospitals. A one standard deviation increase in the number of resident medical professionals per 100 faculty members increased the average efficiency index by 25%. The rest of the hospital and regional variables considered showed coefficients with credible intervals containing the value 0, or a smaller positive coefficient probability in the posterior distribution function (Table 3). For example, variables related to the installed capacity in hospitals (such as emergency physicians and operating beds in intensive care units) or in the region (such as the number of primary care physicians and nurses, emergency physicians or public beds in intensive care units per thousand inhabitants), which are necessary to cope with demand shocks, showed coefficients with credible intervals containing the value 0 or a smaller positive coefficient probability.

When modelling the probability of being on the efficiency frontier, the form of hospital management, the proportion of beds in intensive care units, and the average annual income per household showed coefficients with credible intervals not containing the value 0 or a very high positive coefficient probability in the a posteriori distribution function (Table 3) (Appendix B). On the other hand, the provision of private beds per thousand inhabitants showed a very high negative coefficient probability. The OR of PPP management formulas vs. public hospitals was 42.06. The OR of private hospitals vs. public hospitals was 8.17. A one standard deviation increase in the percentage of beds in Intensive Care Units increased the odds of being on the efficiency frontier by 50%. The odds increased by 6.43 for every one standard deviation increase in the average annual income per household. A one standard deviation increase in private beds per thousand population in the region decreased the odds of being on the efficiency frontier by 63%. The rest of the variables showed coefficients with credible intervals containing the value 0, smaller positive coefficient probability, or small negative coefficient probability in the posterior distribution function as, for example, in this situation, we found in the percentage of occupancy in intensive care units, public beds in the region in intensive care units, or out-of-hospital emergency centers per thousand inhabitants.

## 4. Discussion

This paper is innovative in the econometric field by applying a multilevel zero-one inflated beta regression model to study the hospital and regional variables which are related to both PTE and the probability of being situated on the efficiency frontier. The multilevel zero-one inflated beta regression model fits the characteristic density function of PTE provided by the DEA, and is appropriate for analyzing hierarchically structured information. These two features make the model preferable to other alternatives used hitherto [13,14,15,45,46]. Additionally, multilevel zero-one inflated beta regression models simultaneously predict both the value of PTE and the probability of being on the efficiency frontier as a function of hospital and contextual characteristics. This provides public decision-makers with a higher level of information. Finally, the description of the a posteriori density function using percentiles and probabilities of occurrence in addition to the mean, helped to determine the relationship between each independent and dependent variable. This new approach is an important step in the methodological improvement of efficiency studies in health economics.

The second objective of this article was to analyze the relationship between the installed capacity at both a hospital and regional level and the technical efficiency of SNHS hospitals in order to assess their ability to cope with demand shocks and to evaluate whether hospital efficiency is affected by the hospital provisions in primary care and emergency healthcare resources, which are critical for dealing with these situations.

The results suggest that increasing the number of public beds in hospital intensive care units increases the probability of a hospital being situated on the efficiency frontier. Only one study in Germany assessed the relationship between emergency departments and the technical efficiency of hospitals [16], finding a negative relationship between the dispersion of emergency departments, their caseloads, and hospital efficiency. However, the studies are not comparable due to the different emergency variables used, as well as the different methods and objectives. Contrary to the German study, our results focus on the efficiency frontier, not on the relationship between technical efficiency and the characteristics of hospital emergency departments. The result obtained establishes a link between a higher number of beds in intensive care units and greater odds of being efficient. Further studies should establish whether the association found is robust and analyze possible explanatory hypotheses. The other variables analyzed, related to the ability of the installed capacity to cope with sudden global increases in demand, do not seem to be associated to the level of efficiency of hospitals.

Private ownership and PPP models performed better than public hospitals, both because they achieved higher levels of efficiency and because of the greater odds of being placed on the efficiency frontier. These results are in line with previous research in Spain [14,15,45,46]. Regional health services with centralized governance structures, no management autonomy in public hospitals, and a rigid civil servant labor regime, may partially explain these results. At the regional level, only two variables have shown a relevant probabilistic relationship to the possibility of being on the efficiency frontier: the number of private beds per thousand inhabitants and the average annual income per household. 

The number of private beds per thousand inhabitants decreased by 63% the odds of being positioned on the efficiency frontier. Previous work did not find this relationship significant [47], although the divergence of results is probably due to the different estimation method used. In this sense, the results of this study are more robust when using an econometric specification in line with the DEA technical efficiency density function. The relationship found suggests that a higher private hospital supply at the regional level decreases the odds of SNHS hospitals being on the efficiency frontier, irrespective of ownership structure, i.e., a higher provision of private beds per thousand inhabitants in a region impedes the possibility of SNHS hospitals being on the efficiency frontier in that region.

The regional average annual income per household increased the odds of reaching the efficiency frontier 6.43-fold for every one standard deviation increase in the average annual income per household. This result is in line with previous results [47], and confirms the importance of a region’s income level in improving the possibility of its hospitals’ reaching the efficiency frontier.

There are several limitations to the analysis. The cross-sectional nature of the data limits the robustness of the results, and further studies with panel data are needed to extend this analysis. Secondly, it is possible that there are problems in the specification of the exogenous factors included in the model or that explanatory variables which could modify the impact of the variables analyzed on technical efficiency have been omitted.

As lines of future research, multilevel zero-one inflated beta regression models may be suitable for re-evaluating the second-stage analysis of the efficiency of healthcare organizations for a broad set of hospital and contextual characteristics beyond the two examples developed in this article. The effect of pandemics such as COVID-19, on hospital PTE may also be suitable for modelling where data are available. Pandemics are global demand shocks which test installed capacity at a territorial level, including primary care, emergency departments, and intensive care units, and their ability to cope with them. The relationship between these dimensions and hospital PTE in the context of a pandemic may be relevant for policy makers.

## 5. Conclusions

The statistical model proposed in this paper represents a methodological innovation for hospital efficiency studies in the field of health economics. It integrates the non-parametric frontier approach provided by DEA with a second stage analysis using a multilevel zero-one inflated beta regression model. This allows for the analysis of which variables, at both hospital and contextual level, are related to the degree of hospital efficiency, and the probability of being on the technical efficiency frontier. The method improves PTE modelling when compared to its alternatives.

The proportion of hospital beds in intensive care units is related to a higher probability of being on the efficiency frontier. Other variables related to hospital and regional installed capacity and the ability to cope with demand shocks, such as those generated by a pandemic, were not relevant from a probabilistic point of view.

Hospitals with private or PPP management formulas integrated into the SNHS have higher technical efficiency values, and higher odds of being on the technical efficiency frontier than traditional publicly owned hospitals.

## Figures and Tables

**Table 1:** Input-Output variables of the Spanish National Health System General Hospitals. 2017.

	Mean	Standard Deviation	Maximum	Minimum
Inputs				
Installed beds ^a^	484.03	331.22	1408.00	63.00
Healthcare personnel ^b^	2146.58	1669.66	7947.00	169.50
Non-healthcare personnel ^b^	517.68	456.44	2417.50	20.00
Adjusted purchases and external services ^c^	156,561.26	53,247.90	327,822.98	40,261.73
Outputs				
Total discharges adjusted case by case ^d^	18,833.02	13,578.33	59,811.27	1503.74
Outpatient activity ^e^	31,892.54	20,207.32	108,572.25	5720.43

^a^ Annual average of the installed provision, regardless of whether or not they have actually been in operation throughout the year. ^b^ Includes number of full-time professionals and number of part-time professionals (calculating part-time work at 50%). ^c^ In Euros. To avoid larger hospitals being penalized, the purchases and external services variable was standardized and refers to expenditure per bed. ^d^ Hospital discharges adjusted by applying the average Spanish weight (also known as case-mix index or casuistry index). The average weight is the weighted average of the diagnosis-related group weights of all patients in a given unit, group or a provider. ^e^ Integrates outpatient consultations attended in the hospital itself and in its peripheral specialty centers, patients treated in hospital emergency departments who do not require admission or who have voluntarily discharged themselves, transferred to another health center, or died and subsidiary surgery processes performed under general anesthesia, local anesthesia, or sedation which do not require hospital admission. Source: Prepared by the authors, based on the Statistics on Specialised Healthcare Centres (SIAE) and the Basic Minimum Hospitalisation Data Collective (CMBD-H) from the Spanish Ministry of Health, Consumption, and Social Welfare.

**Table 2:** Regional and hospital variables in the second stage analysis in the evaluation of technical efficiency of Spanish National Health System general hospitals, 2017.

Quantitative Variables	Original Variable				Standardized Variable ^1^			
	Minimum	Maximum	Mean	Standard Deviation	Minimum	Maximum	Mean	Standard Deviation
Hospital variables								
Resident physicians per 100 faculty members	0.00	60.78	23.75	16.08	−1.48	2.30	0.00	1.00
Emergency physicians per 100 faculty members ^a^	4.56	30.58	13.32	4.97	−1.76	3.48	0.00	1.00
% Beds in operation in Intensive Care Units ^b^	0.84	9.36	4.30	1.62	−2.15	3.13	0.00	1.00
% Occupation in Intensive Care Units ^c^	7.12	97.06	56.32	22.27	−2.21	1.83	0.00	1.00
Regional variables								
Public healthcare spending per inhabitant ^d^	1153.43	1710.08	1374.11	145.35	−1.52	2.31	0.00	1.00
Average annual income per household (Thousands of €) ^e^	24,375.00	39,578.00	31,705.28	4170.42	−1.76	1.89	0.00	1.00
Installed private beds per 1000 inhabitants ^f^	0.11	1.06	0.58	0.29	−1.63	1.69	0.00	1.00
Installed public beds per 1000 inhabitants ^f^	2.14	3.77	2.83	0.55	−1.25	1.73	0.00	1.00
Public beds in operation in Intensive Care Units per 1000 inhabitants	0.07	0.09	0.08	0.01	−1.09	1.74	0.00	1.00
Emergency physicians per 1000 inhabitants ^a^	0.16	0.30	0.20	0.03	−1.56	3.89	0.00	1.00
External emergency centers per 1000 inhabitants ^g^	0.01	0.12	0.04	0.03	−1.21	2.73	0.00	1.00
Primary care doctors and nurses per 1000 people allocated ^h^	1.12	2.01	1.44	0.21	−1.52	2.77	0.00	1.00
External emergency personnel per 1000 inhabitants ^i^	0.16	1.07	0.41	0.17	−1.42	3.80	0.00	1.00
Qualitative variables	Number	Percentage						
Exogenous hospital variables								
Hospital type								
Public	147	84.97%						
Private	12	6.94%						
Public-Private Partnership	14	8.09%						

^1^ The variable was rescaled to have a mean of zero and a standard deviation of one. ^a^ Includes professionals from hospital emergency and intensive care units. ^b^ Percentage of beds in operation in intensive care units out of the total number of beds in operation in the hospital. ^c^ (Total stays in intensive care units × 100)/(beds in operation in intensive care units × 365). ^d^ Includes health expenditures financed by the public system, whether produced by its own means or by external means through health care agreements. ^e^ Corresponding to the year prior to the year in which the study was conducted. ^f^ Annual average of the installed allocation, regardless of whether or not they have actually been in operation throughout the year. ^g^ Includes all primary care centers of the National Health System in which urgent health care is provided ^h^ Includes the medical and nursing staff of the primary care services of the National Health System. ^i^ Includes the following professional categories: medicine, nursing, drivers, health emergency technicians, and tele-operators. Source: Prepared by the authors.

**Table 3:** Relationship between categorized independent variables and the technical efficiency of the Spanish National Health System hospitals, 2017.

Variable ^a^	Coefficient	Standard Error	Credibility Interval ^b^		PCP ^c^	NCP ^c^	$\hat{R}$	Bulk-ESS	Tail-ESS	RMAI ^d^
Average Efficiency Index Model										
Intercept	1.00	0.21	0.58	1.40	1.00	0.00	1.00	10,710	8026	
Hospital type										
Public	Reference	Reference	Reference				Reference	Reference	Reference	Reference
Public-Private Partnership	1.45	0.41	0.69	2.32	1.00	0.00	1.00	22,111	13,024	4.27
Private	0.64	0.39	−0.08	1.48	0.96	0.04	1.00	25,642	10,668	1.90
Resident physicians per 100 faculty members	0.22	0.08	0.06	0.38	1.00	0.00	1.00	16,751	12,123	1.25
Emergency physicians per 100 faculty members	0.03	0.08	−0.12	0.18	0.64	0.36	1.00	19,586	12,303	1.03
Occupation in Intensive Care Units (%)	0.00	0.07	−0.14	0.14	0.52	0.48	1.00	20,699	11,979	1.00
Beds in operation in Intensive Care Units (%)	−0.01	0.06	−0.12	0.11	0.45	0.55	1.00	26,946	13,022	0.99
Public healthcare spending per inhabitant	0.29	0.46	−0.59	1.23	0.77	0.23	1.00	6240	5652	1.33
Average annual income per household (thousand €)	0.10	0.48	−0.87	1.09	0.59	0.41	1.00	5279	5106	1.11
Primary care doctors and nurses per 1000 people allocated	0.09	0.50	−0.92	1.10	0.58	0.42	1.00	5313	4869	1.09
Installed private beds per 1000 inhabitants	0.00	0.22	−0.45	0.41	0.51	0.49	1.00	11,941	8845	1.00
External emergency centres per 1000 inhabitants	−0.02	0.52	−1.08	1.00	0.49	0.51	1.00	5423	5204	0.98
External emergency personnel per 1000 inhabitants	−0.10	0.40	−0.91	0.71	0.37	0.63	1.00	5471	5073	0.90
Installed public beds per 1000 inhabitants	−0.11	0.40	−0.91	0.66	0.37	0.63	1.00	7459	6470	0.90
Emergency physicians per 1000 inhabitants	−0.18	0.42	−1.04	0.67	0.30	0.70	1.00	5015	4809	0.83
Public beds in operation in Intensive Care Units per 1000 inhabitants	−0.23	0.42	−1.09	0.63	0.26	0.74	1.00	5455	5540	0.80
Probability of Being Situated on the Efficiency Frontier Model										
Intercept	−3.00	0.44	−3.94	−2.21	0.00	1.00	1.00	12,108	10,551	
Hospital type										
Public	Reference	Reference	Reference				Reference	Reference	Reference	Reference
Public-Private Partnership	3.74	1.22	1.57	6.37	1.00	0.00	1.00	17,875	11,484	42.06
Private	2.10	1.14	−0.07	4.40	0.97	0.03	1.00	20,600	11,977	8.17
Beds in operation in Intensive Care Units (%)	0.41	0.26	−0.09	0.93	0.95	0.05	1.00	25,157	11,536	1.50
Occupation in Intensive Care Units (%)	−0.08	0.35	−0.76	0.61	0.42	0.58	1.00	22,315	12,218	0.93
Resident physicians per 100 faculty members	−0.38	0.43	−1.23	0.48	0.19	0.81	1.00	14,282	12,800	0.68
Emergency physicians per 100 faculty members	−0.43	0.37	−1.18	0.29	0.12	0.88	1.00	20,060	12,905	0.65
Average annual income per household (thousands of €)	1.86	1.38	−0.90	4.62	0.91	0.09	1.00	4774	6823	6.43
Primary care doctors and nurses per 1000 people allocated	1.09	1.27	−1.29	3.72	0.80	0.20	1.00	5435	7190	2.97
Public beds in operation in Intensive Care Units per 1000 inhabitants	0.23	1.03	−1.83	2.29	0.58	0.42	1.00	5450	7804	1.26
Emergency physicians per 1000 inhabitants	0.12	1.12	−2.23	2.29	0.54	0.46	1.00	5126	7031	1.13
External emergency personnel per 1000 inhabitants	0.12	1.13	−2.19	2.30	0.54	0.46	1.00	5716	7733	1.12
Public healthcare spending per inhabitant	−0.28	1.16	−2.57	2.03	0.41	0.59	1.00	5110	6895	0.75
External emergency centres per 1000 inhabitants	−0.43	1.25	−2.97	1.96	0.37	0.63	1.00	5775	8174	0.65
Installed public beds per 1000 inhabitants	−0.84	0.91	−2.63	0.98	0.18	0.82	1.00	6257	8963	0.43
Installed private beds per 1000 inhabitants	−0.98	0.57	−2.19	0.05	0.03	0.97	1.00	17,535	11,127	0.37
Accuracy. Conditional Probability of Inflation one and Random Effect										
Precision (φ)	14.27	1.77	11.01	17.92			1.00	18,753	11,620	
Probability of One-Inflation (θ)	0.97	0.03	0.89	1.00			1.00	19,419	7510	
Random Effect $\left(\sigma_{u_{0}}\right)$	0.57	0.31	0.17	1.34			1.00	3041	4251	

^a^ Quantitative variables are standardized to obtain dimensionless variables with mean 0 and standard deviation 1. ^b^ 2.5th percentile and 97.5th percentile of the posterior distribution function of the coefficient. ^c^ Probability of positive coefficient and probability of negative coefficient in the a posteriori distribution function. ^d^ Ratio of mean efficiency indices: change in the MEI for one standard deviation increase in the quantitative independent variable, or change in the MEI for a category of the qualitative independent variable with respect to the reference category. Source: Prepared by the authors.

**Figure A1:**
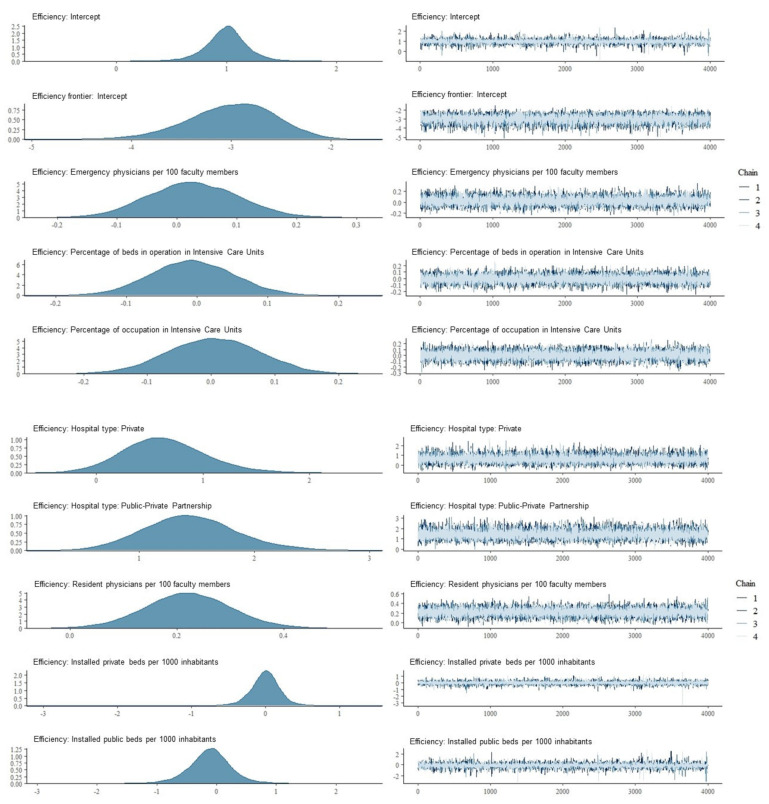

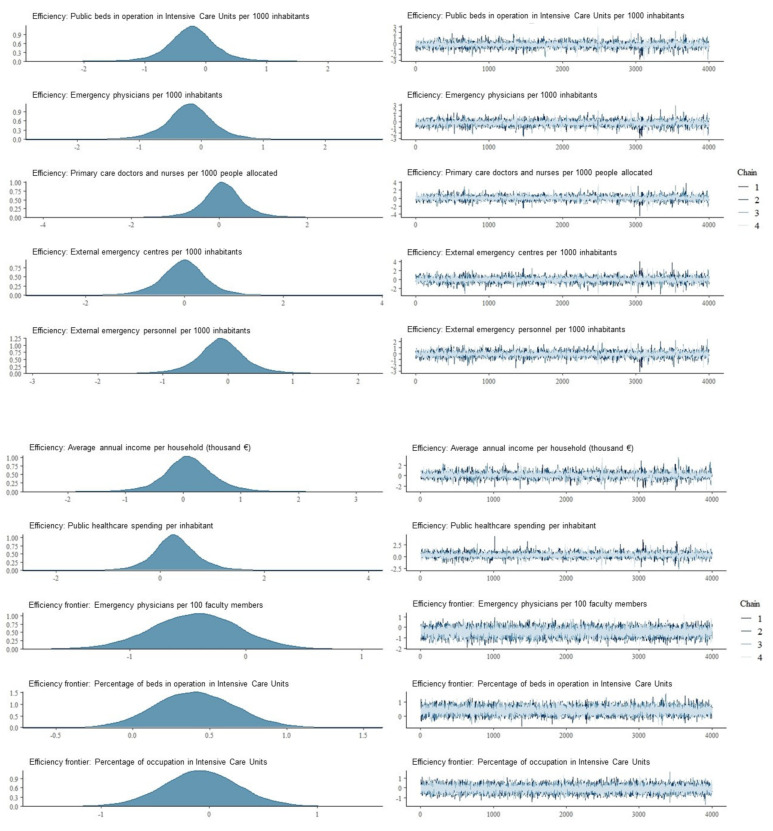

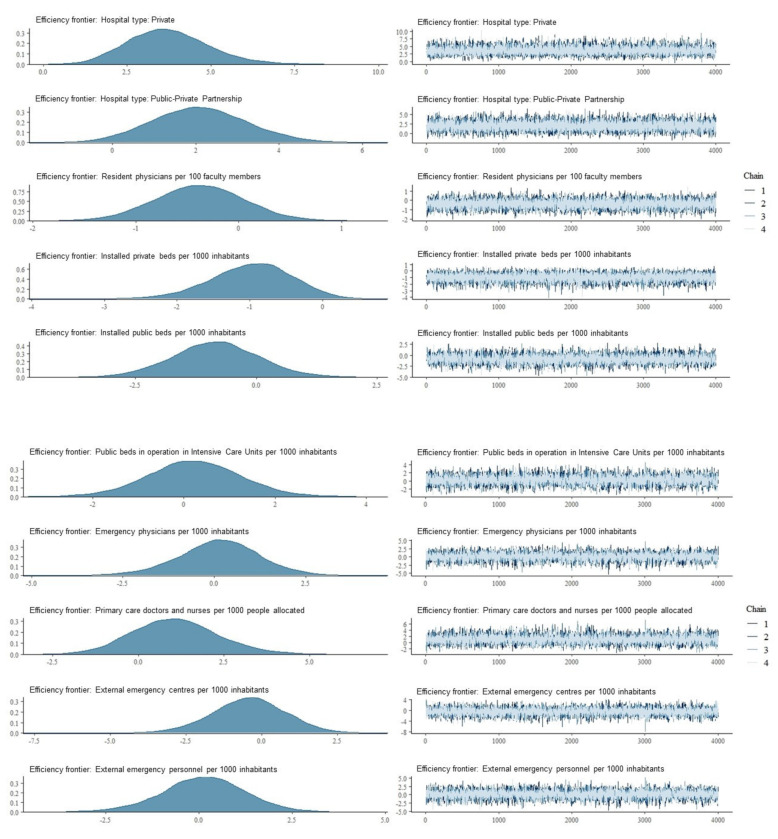

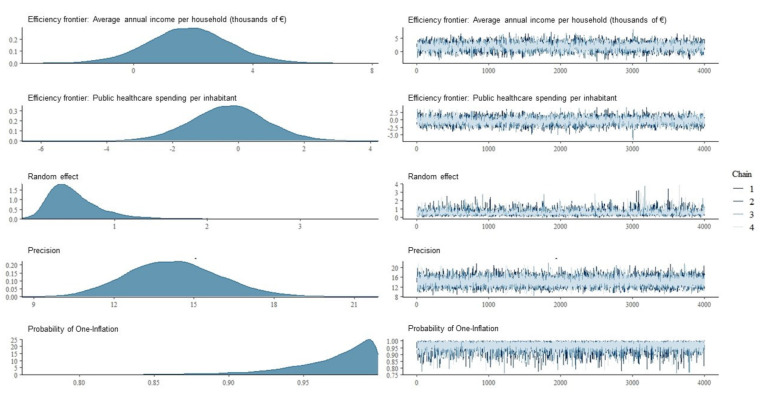
Posterior distribution function of the coefficient of each standardized variable. Variable names starting with “Efficiency” correspond to the distribution of the coefficients in the modelling of the Mean Efficiency Index (MEI). Variable names start-ing with “Efficiency frontier” correspond to the distribution of the coefficients in the mod-elling of the odds of being placed on the efficiency frontier.

## Data Availability

The datasets analysed during the current study are not publicly available and were provided to the authors by the Sub-directorate General of Health Information of the Ministry of Health, Consumption and Social Welfare of the Government of Spain, but are available from the corresponding author on reasonable request.

## Appendix A

(A1)
$$\mathrm{Percentage}\;\mathrm{of}\;\mathrm{emergency}\;\mathrm{and}\;\mathrm{ICU}\;\mathrm{doctors}\;\mathrm{over}\;\mathrm{total}=\left(\frac{\mathrm{Emergency}\;\mathrm{and}\;\mathrm{ICU}\;\mathrm{doctors}}{\mathrm{Total}\;\mathrm{doctors}\;}\right)\times 100$$

(A2)
$$\mathrm{Pressure}\;\mathrm{in}\;\mathrm{the}\;\mathrm{ED}\;\left(\%\right)=\left(\frac{\mathrm{Emergency}\;\mathrm{admissions}}{\;\mathrm{Total}\;\mathrm{hospital}\;\mathrm{admissions}}\right)\times 100$$

(A3)
$$\mathrm{Percentage}\;\mathrm{of}\;\mathrm{ICU}\;\mathrm{bed}\;\mathrm{occupancy}=\left(\frac{\mathrm{ICU}\;\mathrm{stays}}{\mathrm{ICU}\;\mathrm{beds}\;\mathrm{in}\;\mathrm{operation}\;\times 365}\right)\times 100$$

(A4)
$$\mathrm{Percentage}\;\mathrm{of}\;\mathrm{ICU}\;\mathrm{beds}\;\mathrm{over}\;\mathrm{total}\;\mathrm{beds}=\left(\frac{\mathrm{ICU}\;\mathrm{beds}\;\mathrm{in}\;\mathrm{operation}}{\;\mathrm{Total}\;\mathrm{beds}\;\mathrm{in}\;\mathrm{operation}}\right)\times 100$$

(A5)
$$\mathrm{Resident}\;\mathrm{Physicians}\;\mathrm{per}\;100\;\mathrm{doctors}=\left(\frac{\mathrm{Resident}\;\mathrm{Physicians}}{\mathrm{Total}\;\mathrm{doctors}\;}\right)\times 100$$

## Appendix B

The posterior distribution function of the coefficient of each standardized variable is shown below. Variable names starting with “Efficiency” correspond to the distribution of the coefficients in the modelling of the Mean Efficiency Index (MEI). Variable names starting with “Efficiency frontier” correspond to the distribution of the coefficients in the modelling of the odds of being placed on the efficiency frontier. Both PCP (Positive Coefficient Probability) and NPC (Negative Coefficient Probability) are obtained from these distribution functions.
